# Supplementary material for: MicroRNA-221-3p inhibits the inflammatory response of keratinocytes by regulating the DYRK1A/STAT3 signaling pathway to promote wound healing in diabetes
Source: Commun Biol. 2024 Mar 9;7:300. doi: 10.1038/s42003-024-05986-0 (PMC10924844; doi:10.1038/s42003-024-05986-0)
Supplement: Supplementary file 2 — Supplementary Information [file 42003_2024_5986_MOESM2_ESM.pdf]

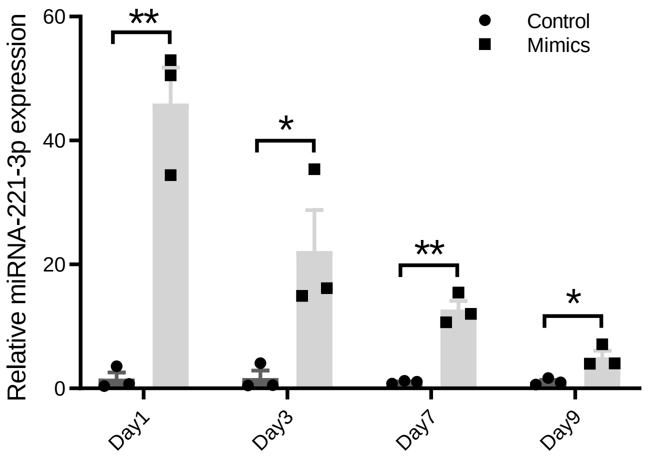

Supplementary Fig. 1. Expression levels of miR-221-3p at the edge of skin wounds in diabetic mice. Summary data showing relative miR-221-3p expression in the edge of skin wounds in a mouse model of diabetes induced by injections of streptozotocin. Total RNA was extracted and levels of miR-221-3p were quantified by qPCR (n=3) with U6 as an internal reference. All data are presented as mean ± SEM; $^{*}P < 0.05$, $^{**}P < 0.01$.

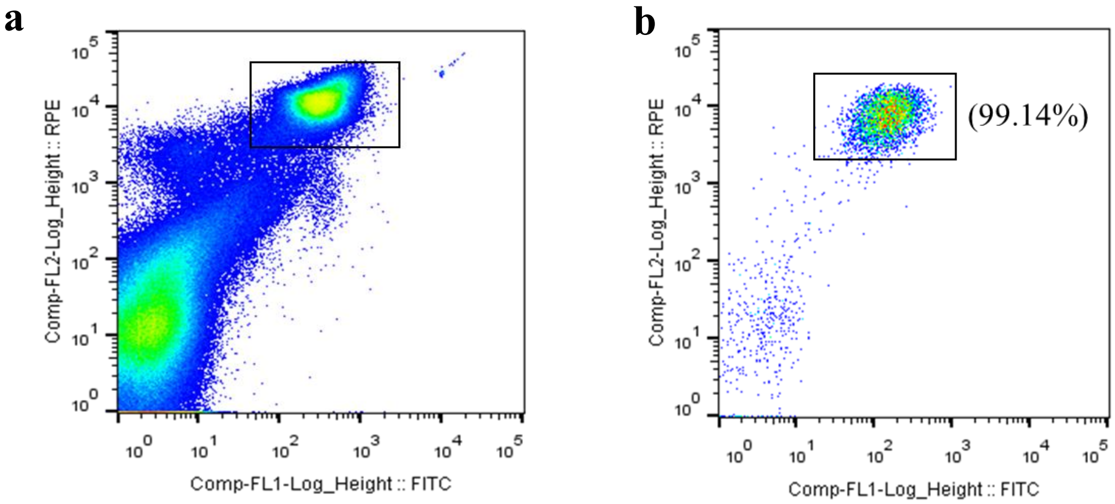

Supplementary Fig. 2. Separation and validation of neutrophils by flow cytometry. CD66b- and CD16-positive cells were sorted from human peripheral blood by flow cytometry. **a**, Double-stained cells representing neutrophils are shown in the framed section of the scatter plot and were collected separately. Harvested neutrophils were validated using flow cytometry. **b**, Results show that over 99% of harvested cells were both CD66b- and CD16-positive.

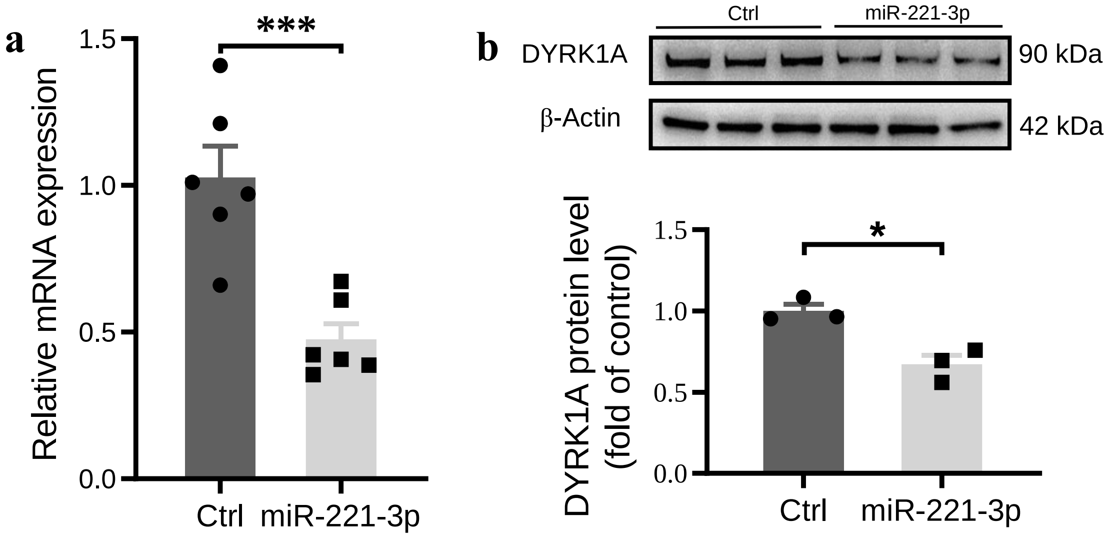

Supplementary Fig. 3. Effects of miR-221-3p on the mRNA and protein expression levels of DYRK1A. HaCaT cells were transfected with miR-221-3p mimic or miRNA mimic negative control (Ctrl). Cells were collected 48 h after transfection. **a**, Summary data showing the relative expression levels of *DYRK1A* mRNA quantified by qPCR. *ACTB* was used as the internal reference (n = 6). **b**, Representative blots and summary data showing DYRK1A protein expression levels. β-Actin (lower panel) was used as the internal control (n = 3). All data are presented as mean ± SEM; $^{*}P < 0.05$, $^{***}P < 0.001$.

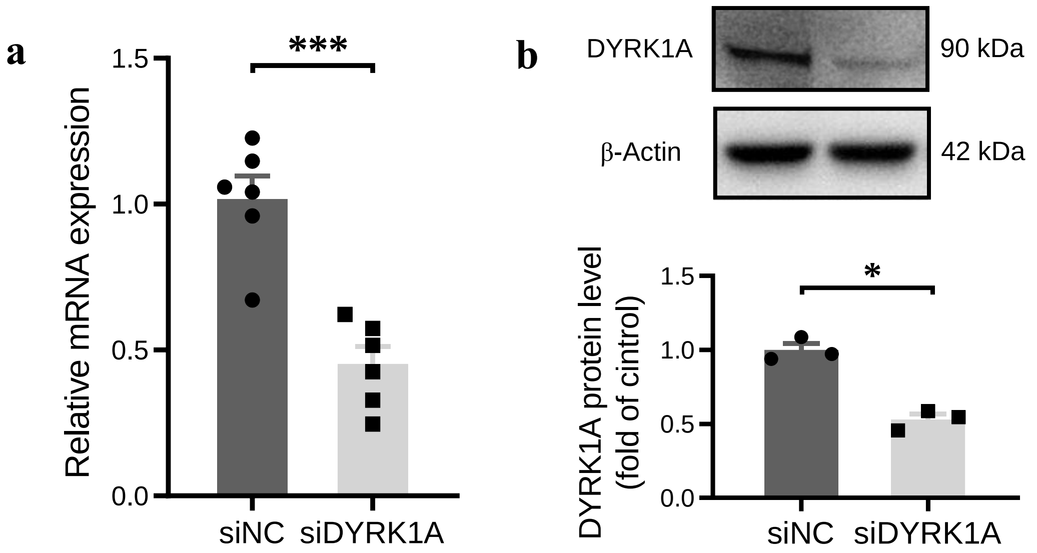

Supplementary Fig. 4. Effects of DYRK1A-specific siRNA on expression levels of DYRK1A. HaCaT cells were transfected with DYRK1A-specific siRNA (siDYRK1A) or negative control (siNC). Cells were collected 48 h after transfection. **a**, Summary data showing the relative expression levels of *DYRK1A* mRNA quantified by qPCR. *ACTB* was used as the internal reference (n = 6). **b**, Representative blots and summary data showing DYRK1A protein expression levels. β-Actin (lower panel) was used as the internal control. All data are presented as mean ± SEM (n = 3); $^{*}P < 0.05$, $^{***}P < 0.001$

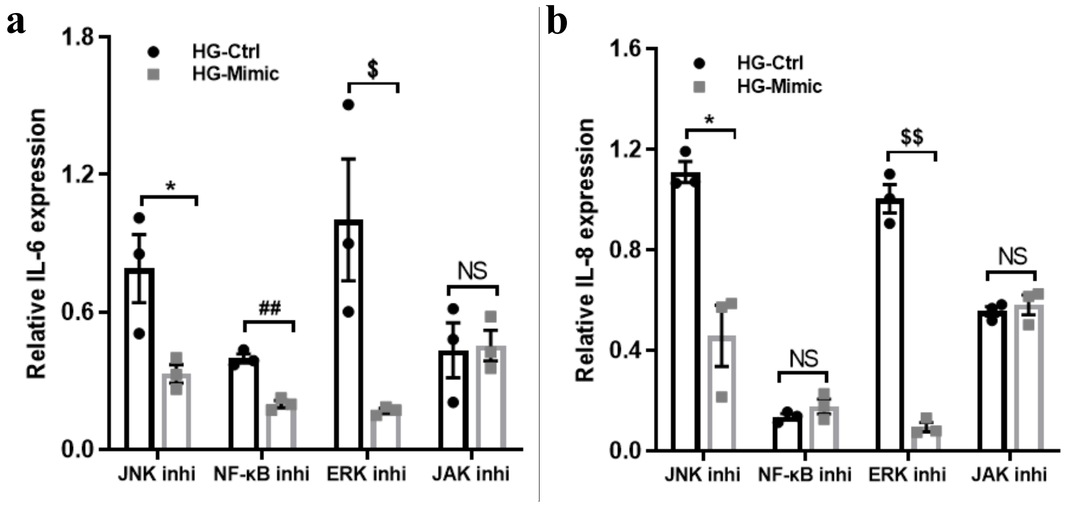

Supplementary Fig. 5. Signaling pathways influenced by miR-221-3p with various inhibitors. HaCaT cells were cultured in high-glucose medium and pretreated with various inhibitors before cells were transfected with miR-221-3p mimic (HG-Mimic) or negative control (HG-Ctrl). *IL-6* (**a**) and *IL-8* (**b**) mRNA expression levels were examined by qPCR. All numeric data are presented as mean ± SEM (n = 3). $^{*}P < 0.05$ for HG-Mimic vs. HG-Ctrl pretreated with JNK inhi; $^{\#\#}P < 0.01$ for HG-Mimic vs. HG-Ctrl pretreated with NF-κB inhi; $^{\$}P < 0.05$, $^{\$\$}P < 0.01$ for HG-Mimic vs. HG-Ctrl pretreated with ERK inhi; NS: No significance. JNK inhi represents, c-Jun N-terminal kinase inhibitor; NF-κB inhi, nuclear factor-kappa B inhibitor; ERK inhi, extracellular signal-regulated kinase inhibitor; JAK inhi, janus kinase inhibitor.

Supplementary Fig. 6. Experimental Schematic illustration for mice.

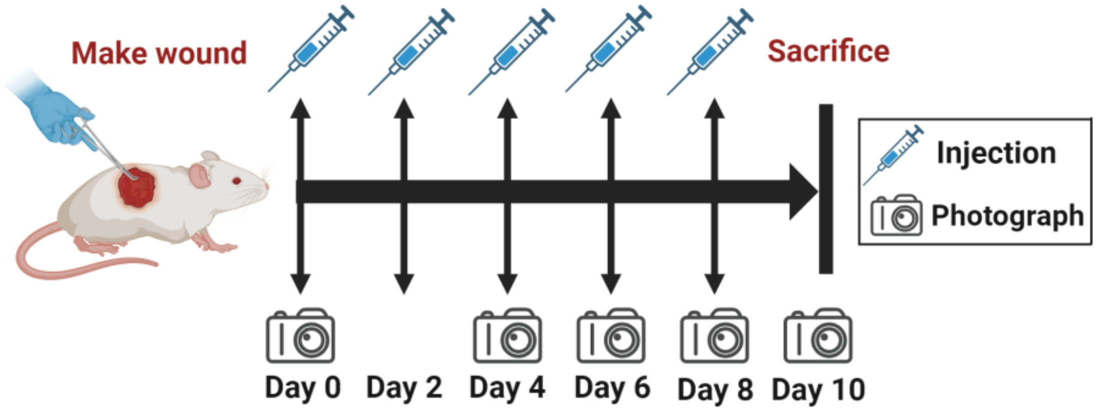

Created excisional wounds, the size of the wound area was photographed and measured every other day. Created with BioRender.com.

Supplementary Fig. 7. Images of uncropped blots

Figure 4e

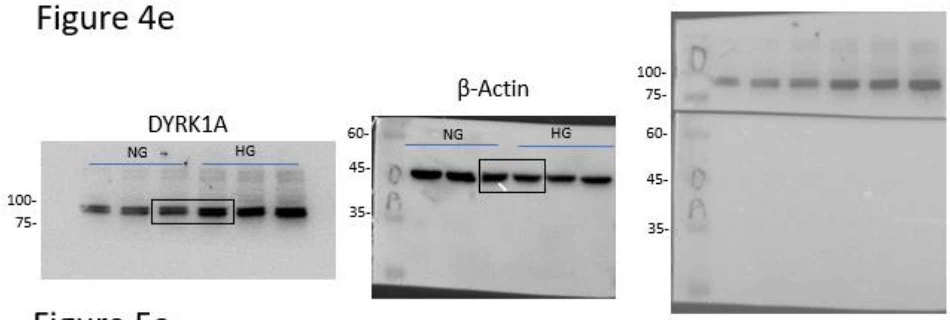

Figure 5a

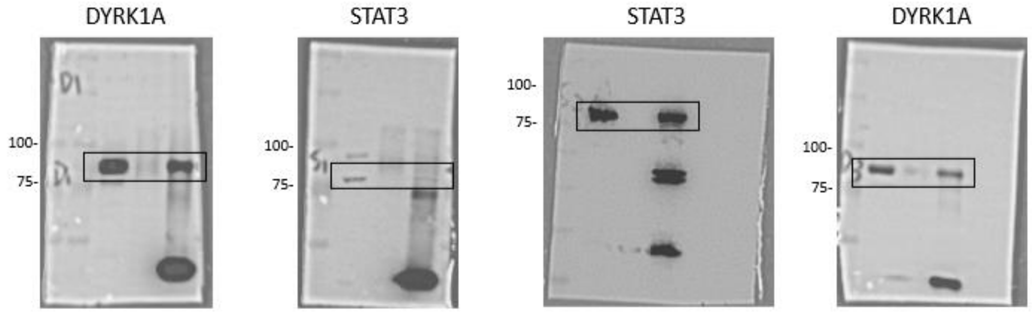

Figure 5b

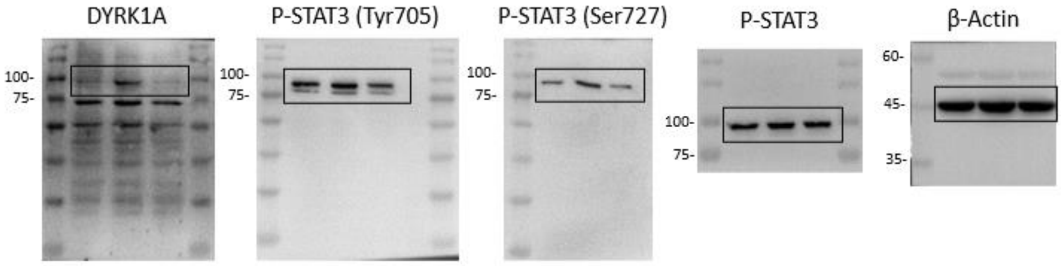

Figure 5c

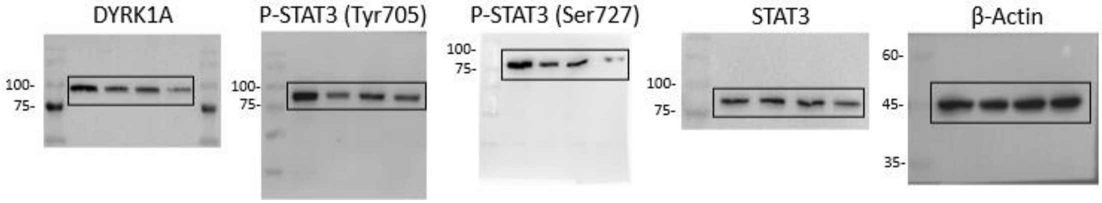

Figure 6d

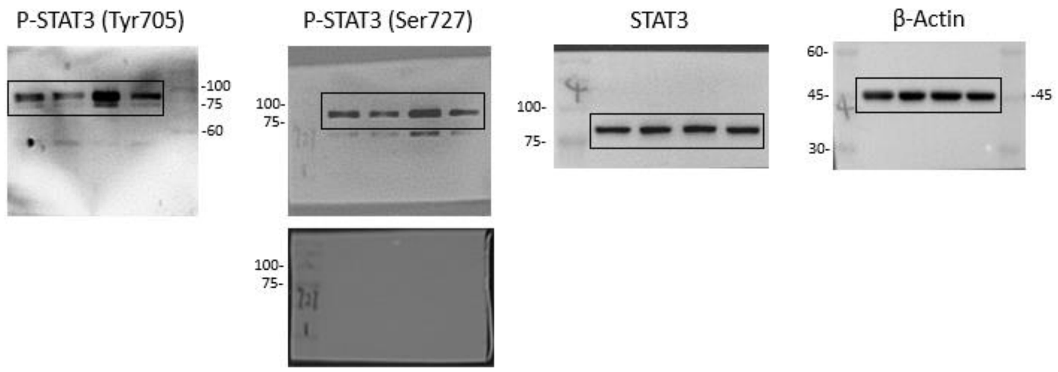

Figure 8d

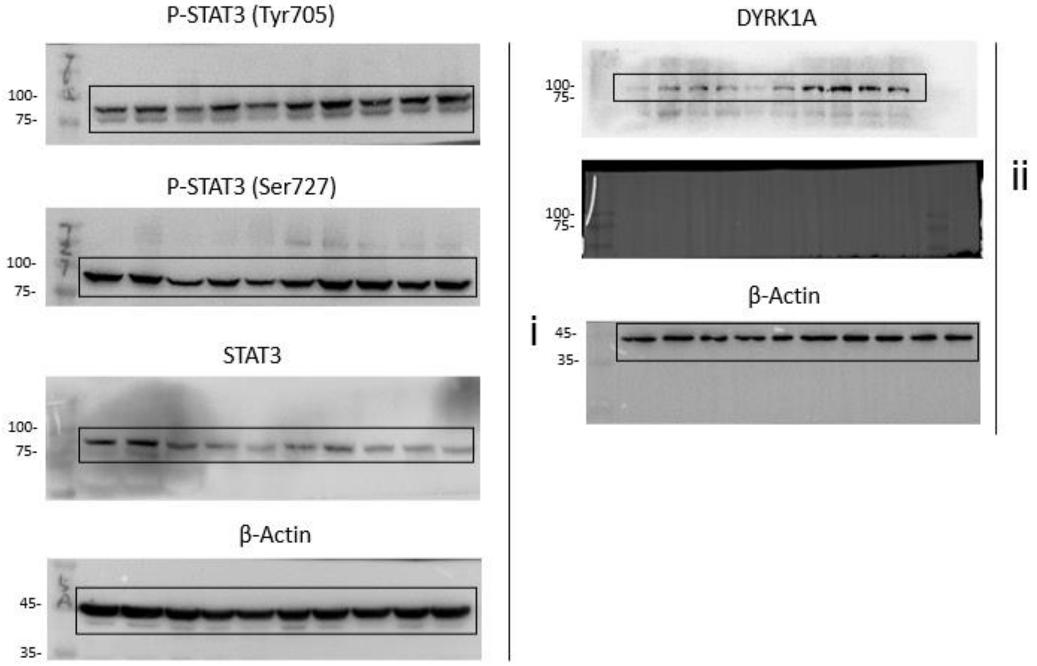

Figure 9b

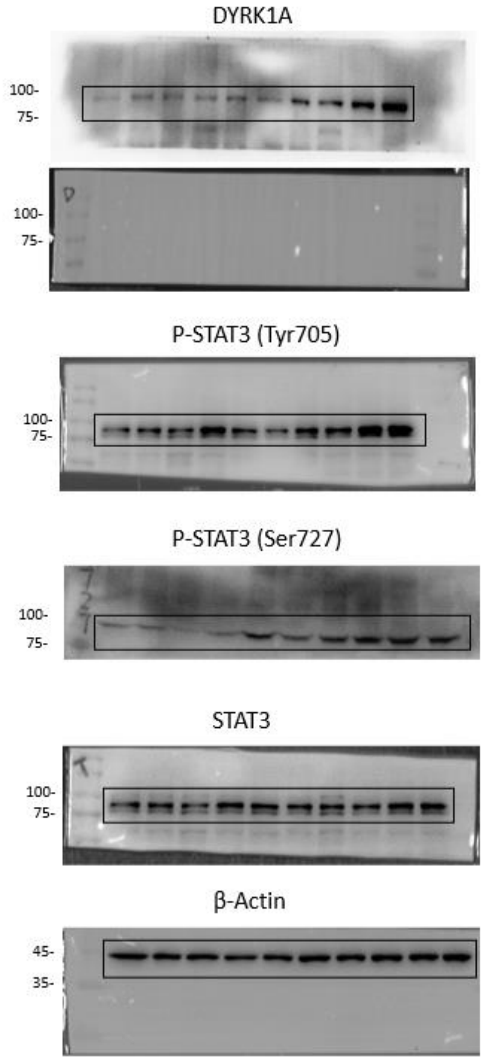

Figure 9d

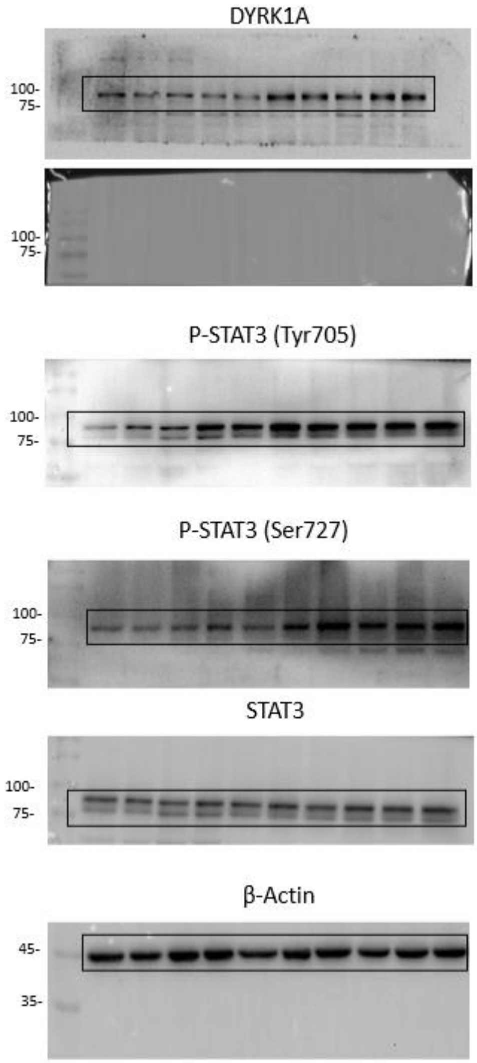

Supplementary Figure 3b

DYRK1A

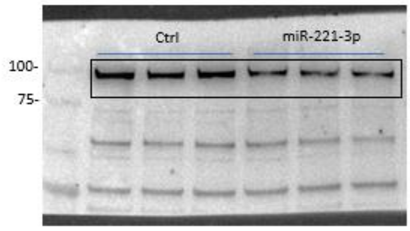

β-Actin

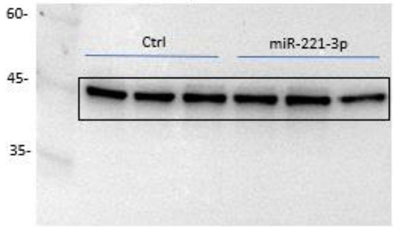

Supplementary Figure 4b

DYRK1A

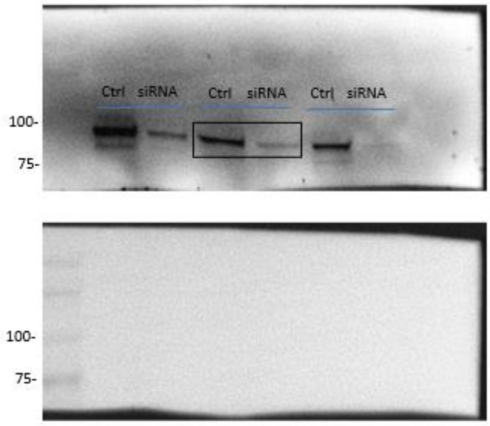

β-Actin

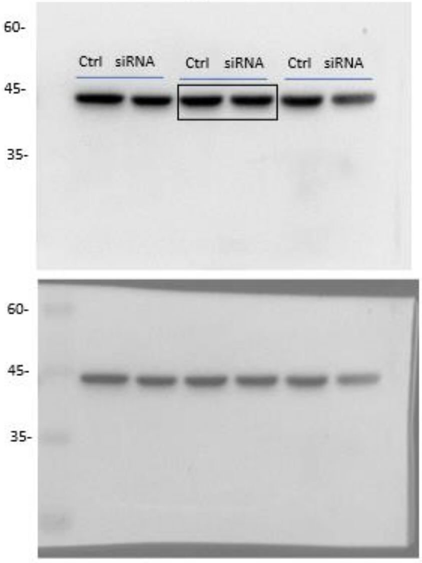

Supplementary Table 1. KEGG analysis results for pathways upregulated in HG-Ctrl vs. NG-Ctrl

Term	ES	NES	P value	FDR	Gene set size	Matched size
Salmonella infection (hsa05132)	0.617878	2.697636	0	0	84	81
NF-kappa B signaling pathway (hsa04064)	0.591044	2.64811	0	0	93	89
IL-17 signaling pathway (hsa04657)	0.579353	2.550637	0	0	93	85
AGE-RAGE signaling pathway in diabetic complications (hsa04933)	0.55659	2.526425	0	0	99	99
Rheumatoid arthritis (hsa05323)	0.542333	2.41917	0	0	86	83
TNF signaling pathway (hsa04668)	0.513418	2.343837	0	0	110	110
Malaria (hsa05144)	0.581927	2.195436	0	0.000488	49	40
Th17 cell differentiation (hsa04659)	0.481378	2.192783	0	0.000651	105	98
Chagas disease (American trypanosomiasis) (hsa05142)	0.478309	2.171763	0	0.000586	101	95
Ribosome biogenesis in eukaryotes (hsa03008)	0.49443	2.166477	0	0.000533	81	76
Amoebiasis (hsa05146)	0.477303	2.160324	0	0.000488	95	90
Legionellosis (hsa05134)	0.544806	2.15988	0	0.000601	55	53
Toll-like receptor signaling pathway (hsa04620)	0.485004	2.125733	0	0.000697	102	87
Cytokine-cytokine receptor interaction (hsa04060)	0.412749	2.11386	0	0.000781	292	241
Osteoclast differentiation (hsa04380)	0.44929	2.102037	0	0.000854	125	119
C-type lectin receptor signaling pathway (hsa04625)	0.465292	2.089448	0	0.000919	104	96
Bladder cancer (hsa05219)	0.548093	2.08716	0.003012	0.000868	41	40
MAPK signaling pathway (hsa04010)	0.394195	2.077777	0	0.000925	295	291
Colorectal cancer (hsa05210)	0.467134	2.041405	0	0.001562	86	86
Pertussis (hsa05133)	0.478338	2.026339	0	0.001674	76	69
Proteoglycans in cancer (hsa05205)	0.404713	2.023673	0	0.001775	198	190
Kaposi sarcoma-associated herpesvirus infection (hsa05167)	0.408269	1.9995	0	0.002207	186	171
Focal adhesion (hsa04510)	0.393254	1.998613	0	0.002197	199	197
Antifolate resistance (hsa01523)	0.575605	1.99348	0	0.002343	31	30
Human T-cell leukemia virus 1 infection (hsa05166)	0.382151	1.980668	0	0.002553	253	247
Chemokine signaling pathway (hsa04062)	0.396644	1.964197	0	0.00282	187	168
Small cell lung cancer (hsa05222)	0.435095	1.951578	0	0.002999	93	92
Hematopoietic cell lineage (hsa04640)	0.457836	1.950655	0	0.00303	94	83
T cell receptor signaling pathway (hsa04660)	0.427952	1.95047	0	0.002929	101	95
Th1 and Th2 cell differentiation (hsa04658)	0.431447	1.948149	0	0.002897	90	84
Inflammatory bowel disease (IBD) (hsa05321)	0.489513	1.943828	0	0.002929	63	54
MicroRNAs in cancer (hsa05206)	0.407795	1.93845	0	0.003018	150	148
Pathways in cancer (hsa05200)	0.342597	1.931588	0	0.003044	523	494

ErbB signaling pathway (hsa04012)	0.435117	1.910685	0	0.003682	85	85
Hepatitis B (hsa05161)	0.40338	1.904896	0	0.003688	143	134
JAK-STAT signaling pathway (hsa04630)	0.399918	1.898256	0	0.003747	162	138
Prion diseases (hsa05020)	0.535731	1.897117	0.002667	0.003648	34	31
Fluid shear stress and atherosclerosis (hsa05418)	0.394967	1.893263	0	0.003755	136	129
Prostate cancer (hsa05215)	0.422828	1.884065	0	0.004003	97	95
Hippo signaling pathway - multiple species (hsa04392)	0.536519	1.877469	0.002882	0.004286	29	29
Measles (hsa05162)	0.406234	1.862332	0	0.004742	131	116
PI3K-Akt signaling pathway (hsa04151)	0.347513	1.862092	0	0.004632	353	330
Glycosaminoglycan biosynthesis - chondroitin sulfate / de... (hsa00532)	0.59015	1.856538	0.01039	0.004704	20	20
EGFR tyrosine kinase inhibitor resistance (hsa01521)	0.423749	1.851198	0	0.00486	79	79
Melanoma (hsa05218)	0.428815	1.836401	0	0.005518	72	71
Epithelial cell signaling in Helicobacter pylori infection (hsa05120)	0.4292	1.825469	0.003448	0.006689	68	67
Cytosolic DNA-sensing pathway (hsa04623)	0.452121	1.821313	0	0.006834	62	51
Chronic myeloid leukemia (hsa05220)	0.422744	1.798552	0.00339	0.008089	76	76
Cellular senescence (hsa04218)	0.371356	1.793622	0	0.008123	158	155
NOD-like receptor signaling pathway (hsa04621)	0.369667	1.792004	0	0.008231	167	154
Influenza A (hsa05164)	0.361853	1.77414	0	0.009012	169	153
Acute myeloid leukemia (hsa05221)	0.431818	1.76675	0	0.009358	66	66
Transcriptional misregulation in cancer (hsa05202)	0.358841	1.760296	0	0.009871	185	177
Human cytomegalovirus infection (hsa05163)	0.353705	1.754502	0	0.010295	223	206
Renal cell carcinoma (hsa05211)	0.407407	1.749709	0	0.010565	69	69
Leishmaniasis (hsa05140)	0.409877	1.727764	0	0.012401	70	67
RIG-I-like receptor signaling pathway (hsa04622)	0.416257	1.72362	0.006623	0.012422	70	59
Relaxin signaling pathway (hsa04926)	0.361802	1.723208	0	0.012245	130	124
Type II diabetes mellitus (hsa04930)	0.458751	1.716285	0.003236	0.012789	46	43
Breast cancer (hsa05224)	0.35482	1.704378	0	0.013604	147	142
Adherens junction (hsa04520)	0.39489	1.698881	0	0.01414	72	72
Endocrine resistance (hsa01522)	0.370601	1.691763	0	0.01497	96	96
ECM-receptor interaction (hsa04512)	0.395438	1.689869	0	0.01498	82	80
RNA transport (hsa03013)	0.343498	1.685532	0	0.01529	166	161
Regulation of lipolysis in adipocytes (hsa04923)	0.407451	1.661947	0.003049	0.018046	54	51
Hypertrophic cardiomyopathy (HCM) (hsa05410)	0.378183	1.659645	0	0.018069	85	81
Epstein-Barr virus infection (hsa05169)	0.330675	1.655546	0	0.018406	198	188
B cell receptor signaling pathway (hsa04662)	0.38669	1.642514	0.00692	0.020007	70	70
Glycosaminoglycan biosynthesis - heparan sulfate / heparin (hsa00534)	0.506629	1.628205	0.011364	0.022204	24	24
TGF-beta signaling pathway (hsa04350)	0.372684	1.615268	0	0.024173	84	83
Parathyroid hormone synthesis, secretion and action	0.352127	1.604853	0	0.025763	106	105

(hsa04928)						
Hippo signaling pathway (hsa04390)	0.333242	1.596742	0	0.026694	154	149
Shigellosis (hsa05131)	0.387282	1.595151	0.003436	0.02665	65	65
Tuberculosis (hsa05152)	0.317798	1.554895	0	0.03525	175	161
African trypanosomiasis (hsa05143)	0.447037	1.554707	0.023055	0.034812	34	28
Ras signaling pathway (hsa04014)	0.300161	1.544956	0	0.037073	232	220
Gastric cancer (hsa05226)	0.321873	1.543126	0.004566	0.037124	148	142
Pancreatic cancer (hsa05212)	0.354471	1.508058	0.02439	0.047405	75	75
Herpes simplex infection (hsa05168)	0.30625	1.50659	0	0.047155	183	169
Toxoplasmosis (hsa05145)	0.326163	1.503586	0.003745	0.047657	111	105
Glioma (hsa05214)	0.356422	1.50081	0.020408	0.047814	71	70
Rap1 signaling pathway (hsa04015)	0.291081	1.491782	0	0.050273	206	202
Natural killer cell mediated cytotoxicity (hsa04650)	0.325254	1.489185	0.004	0.050325	127	110
HIF-1 signaling pathway (hsa04066)	0.322282	1.467747	0.024194	0.058416	100	97
Hepatocellular carcinoma (hsa05225)	0.294558	1.461636	0.004505	0.060076	165	158
Dilated cardiomyopathy (DCM) (hsa05414)	0.329996	1.446716	0.02214	0.065804	89	86
VEGF signaling pathway (hsa04370)	0.350207	1.416022	0.043077	0.080144	59	58
Neurotrophin signaling pathway (hsa04722)	0.298053	1.394199	0.017794	0.090643	119	118
Wnt signaling pathway (hsa04310)	0.287049	1.382128	0.008511	0.096334	146	138
Regulation of actin cytoskeleton (hsa04810)	0.270697	1.378645	0.021053	0.097398	213	208
Thyroid hormone signaling pathway (hsa04919)	0.293402	1.367315	0.028226	0.103029	116	113
Human papillomavirus infection (hsa05165)	0.255925	1.366491	0	0.102376	339	323
Endometrial cancer (hsa05213)	0.332688	1.362559	0.042345	0.103934	58	58
FoxO signaling pathway (hsa04068)	0.288148	1.361053	0.003984	0.10409	132	130
Longevity regulating pathway - multiple species (hsa04213)	0.332405	1.358025	0.039088	0.103782	62	60
Protein processing in endoplasmic reticulum (hsa04141)	0.280313	1.353418	0.022624	0.105693	164	159
Hepatitis C (hsa05160)	0.277794	1.292736	0.048193	0.150386	131	120
Axon guidance (hsa04360)	0.255058	1.265913	0.040541	0.173965	175	174

Abbreviations: ES, enrichment score; NES, normalized enrichment score; FDR, false discovery rate.

Supplementary Table 2. KEGG analysis for pathways downregulated in HG-mimic vs. HG-Ctrl

Term	ES	NES	P value	FDR	Gene set size	Matched size
Systemic lupus erythematosus (hsa05322)	-0.88694	-3.2502	0	0	136	99
Alcoholism (hsa05034)	-0.78553	-2.99062	0	0	187	150
Viral carcinogenesis (hsa05203)	-0.57782	-2.28607	0	0	204	187
Adherens junction (hsa04520)	-0.6285	-2.14767	0	0.000201	71	69
Herpes simplex virus 1 infection (hsa05168)	-0.4392	-1.88145	0	0.007547	498	428
Phosphatidylinositol signaling system (hsa04070)	-0.52585	-1.87144	0	0.00805	97	89
Transcriptional misregulation in cancer (hsa05202)	-0.46486	-1.80447	0	0.018994	192	160
Inositol phosphate metabolism (hsa00562)	-0.51242	-1.75503	0	0.030524	73	69
Necroptosis (hsa04217)	-0.46142	-1.72485	0	0.040855	159	126
MicroRNAs in cancer (hsa05206)	-0.42888	-1.64555	0.00137	0.086541	161	151
TGF-beta signaling pathway (hsa04350)	-0.46257	-1.6273	0	0.094675	94	81
Pancreatic secretion (hsa04972)	-0.48951	-1.62709	0	0.087547	101	58
Lysine degradation (hsa00310)	-0.47818	-1.6075	0.012924	0.100772	63	61
Shigellosis (hsa05131)	-0.39464	-1.57978	0.00137	0.124243	245	234
Hedgehog signaling pathway (hsa04340)	-0.49113	-1.56945	0.005025	0.128302	50	47
Axon guidance (hsa04360)	-0.40358	-1.56423	0	0.1262	182	165
Aldosterone-regulated sodium reabsorption (hsa04960)	-0.56048	-1.54911	0.027397	0.138253	37	25
Measles (hsa05162)	-0.41727	-1.53446	0.001511	0.149354	139	110
Bile secretion (hsa04976)	-0.46855	-1.49038	0.022034	0.209408	90	47
Focal adhesion (hsa04510)	-0.3775	-1.47396	0.009943	0.227421	201	177
Type II diabetes mellitus (hsa04930)	-0.48316	-1.46174	0.036066	0.239999	46	36
AMPK signaling pathway (hsa04152)	-0.39684	-1.45704	0.01072	0.238315	120	107
JAK-STAT signaling pathway (hsa04630)	-0.38963	-1.44287	0.013657	0.257274	162	113
Thyroid hormone synthesis (hsa04918)	-0.44274	-1.44064	0.038128	0.251169	75	51
Ubiquitin mediated proteolysis (hsa04120)	-0.3749	-1.42241	0.024963	0.279941	139	134
Thyroid hormone signaling pathway (hsa04919)	-0.38143	-1.4097	0.023704	0.296705	121	104
Rap1 signaling pathway (hsa04015)	-0.35612	-1.39734	0.007519	0.312811	210	174
Wnt signaling pathway (hsa04310)	-0.36982	-1.39385	0.018154	0.299873	160	129
ECM-receptor interaction (hsa04512)	-0.40336	-1.37967	0.045977	0.32344	88	68
Proteoglycans in cancer (hsa05205)	-0.34886	-1.35298	0.029787	0.37071	202	176
PI3K-Akt signaling pathway (hsa04151)	-0.31127	-1.28032	0.023904	0.456854	354	274

Abbreviations: ES, enrichment score; NES, normalized enrichment score; FDR, false discovery rate.

Supplementary Table 3. KEGG analysis for pathways downregulated in NG-mimic vs. NG-Ctrl

Term	ES	NES	P value	FDR	Gene set size	Matched size
Systemic lupus erythematosus (hsa05322)	-0.73621	-2.6705	0	0	136	98
Alcoholism (hsa05034)	-0.58178	-2.30036	0	0	187	152
Adherens junction (hsa04520)	-0.60673	-2.0973	0	0	71	70
Viral carcinogenesis (hsa05203)	-0.47379	-1.89179	0	0.009013	204	186
Inositol phosphate metabolism (hsa00562)	-0.50814	-1.74623	0	0.050282	73	69
Phosphatidylinositol signaling system (hsa04070)	-0.4781	-1.73047	0	0.050968	97	90
Herpes simplex virus 1 infection (hsa05168)	-0.37895	-1.63011	0	0.119081	498	426
Type II diabetes mellitus (hsa04930)	-0.53211	-1.61959	0.012195	0.116838	46	35
Pancreatic secretion (hsa04972)	-0.47437	-1.59183	0.011142	0.134948	101	61
Thyroid hormone synthesis (hsa04918)	-0.49216	-1.58791	0.017327	0.126095	75	52
AGE-RAGE signaling pathway in diabetic complications (hsa04933)	-0.43466	-1.58336	0.005831	0.119759	100	93
Measles (hsa05162)	-0.42711	-1.57919	0	0.114495	139	109
Malaria (hsa05144)	-0.54802	-1.5746	0.02046	0.111503	50	28
Focal adhesion (hsa04510)	-0.39111	-1.55073	0	0.126686	201	182
Th17 cell differentiation (hsa04659)	-0.42248	-1.51884	0.006042	0.154602	107	85
Taste transduction (hsa04742)	-0.49746	-1.51823	0.026764	0.146171	86	39
Shigellosis (hsa05131)	-0.35981	-1.49342	0.004082	0.167608	245	233
AMPK signaling pathway (hsa04152)	-0.39917	-1.46251	0.01791	0.202008	120	109
Insulin resistance (hsa04931)	-0.39997	-1.44991	0.014837	0.210935	108	98
MicroRNAs in cancer (hsa05206)	-0.36781	-1.43606	0.007092	0.221218	161	155
Arrhythmogenic right ventricular cardiomyopathy (hsa05412)	-0.42635	-1.4079	0.045333	0.249443	77	61
JAK-STAT signaling pathway (hsa04630)	-0.37239	-1.39133	0.023669	0.246083	162	112
Cortisol synthesis and secretion (hsa04927)	-0.4229	-1.37269	0.034667	0.26007	65	47
Inflammatory mediator regulation of TRP channels (hsa04750)	-0.38542	-1.36016	0.025496	0.272729	98	81
Calcium signaling pathway (hsa04020)	-0.34776	-1.35803	0.022642	0.267018	240	177
NOD-like receptor signaling pathway (hsa04621)	-0.35123	-1.35065	0.022293	0.272578	180	143
RNA transport (hsa03013)	-0.34367	-1.34266	0.016611	0.277791	166	155
Leukocyte transendothelial migration (hsa04670)	-0.36968	-1.33864	0.045181	0.268299	114	95
Rap1 signaling pathway (hsa04015)	-0.33924	-1.31941	0.013793	0.296257	210	175
Proteoglycans in cancer (hsa05205)	-0.33081	-1.30546	0.015038	0.308494	202	178
Transcriptional misregulation in cancer (hsa05202)	-0.32896	-1.29073	0.032895	0.320054	192	162
Ubiquitin mediated proteolysis (hsa04120)	-0.33849	-1.27574	0.043011	0.326462	139	136
Regulation of actin cytoskeleton (hsa04810)	-0.31459	-1.24248	0.045614	0.374634	218	190

Abbreviations: ES, enrichment score; NES, normalized enrichment score; FDR, false discovery rate.

Supplementary Table 4. Genes enriched in the JAK-STAT signaling pathway

**HG-Ctrl vs. NG-Ctrl**	**HG-mimic vs. HG-Ctrl**	**HG-mimic vs. HG-Ctrl**
IL7R	LEPR	PIM1
EGFR	IL6ST	STAM
LIF	JAK2	IL7R
IL2RG	SOS1	CNTF
OSMR	CCND1	IFNGR2
CSF3	IFNAR1	OSMR
PIK3CD	STAT6	CISH
PDGFRA	CSF3R	IL22RA1
IFNL3	STAM2	PIAS2
IL20	SOCS2	PIAS1
IFNA1	PIAS3	AOX1
IL27RA	IL7R	EGFR
IFNGR2	IFNLR1	CSF3R
IL12RB2	PTPN11	SOCS7
PTPN11	RAF1	CREBBP
FHL1	PIK3CA	IFNAR2
IFNE	AOX1	EP300
EP300	IL13RA1	IL20RB
IL4R	SOS2	JAK1
IL2RA	CNTF	STAT6
SOCS6	LIFR	JAK3
IL13RA2	STAT1	MYC
SOCS7	SOCS7	MPL
MCL1	MPL	IFNLR1
CREBBP	STAM	SOCS2
IFNL2	PIK3CB	PDGFA
IFNA14	AKT3	PDGFRA
IFNA7	PIK3R1	STAT2
PRL	IFNAR2	MTOR
OSM	PIAS2	STAT3
IL22RA2	OSMR	CCND2
IL10RA	MYC	
IL5RA	PDGFRA	
PDGFRB	EGFR	
CSF3R	PIAS1	
SOCS5	CREBBP	
PIK3CA	JAK1	
IL11	EP300	
SOCS4	PDGFA	
SOCS2	STAT3	
CDKN1A	MTOR	

MPL	IL20RB	
JAK2	STAT2	
PIK3CB	CCND2	

Supplementary Table 5. Primers and oligos used in this study

**qPCR primer**	**Forward sequence (5′–3′)**	**Reverse sequence (5′–3′)**
hs-IL-1β	GCCAGTGAAATGATGGCTTATT	AGGAGCACTTCATCTGTTTAGG
hs-IL-6	ACTCACCTCTTCAGAACGAATTG	CCATCTTTGGAAGGTTCAGGTTG
hs-IL-8	TTTTGCCAAGGAGTGCTAAAGA	AACCCTCTGCACCCAGTTTTC
hs-TNF-α	CCTCTCTCTAATCAGCCCTCTG	GAGGACCTGGGAGTAGATGAG
hs-DYRK1A	CAGGTGCGTCAGCAATTTCC	ATGCATTCTGTTGAGGGGCT
β-actin	CATGTACGTTGCTATCCAGGC	CTCCTTAATGTCACGCACGAT
ms-IL-1β	ACTGACTGGGACCCTCAAGT	GCAAGACGTGTACGAGTGGT
ms-IL-6	TGGTCTTCTGGAGTACCATAGC	TGTGACTCCAGCTTATCTCTTGG
ms-IL-8	CCTGATGCTCCATGGGTGAA	ACAGAAGCTTCATTGCCGGT
ms-TNF-α	GATCGGTCCCCAAAGGGATG	CCACTTGGTGGTTTGTGAGTG
iNOS	ATGCGAAAGGTCATGGCTTC	CCCAAATGTGCTTGTCACCA
CD86	CTGCACGTCTAAGCAAGGTC	CAGAACACACACAACGGTCA
IL-10	ATTTGAATTCCCTGGGTGAG	CCTTGGTCTTGGAGCTTATT
CD206	CTGAGTAGTCGCAGTGGTTG	GGACTTCCTGGTAGCCAGTT
ms-DYRK1A	TTGCACCGTCGTTCTCATTC	CACCTGCTGGTCACTTATGC
ms-18S	ACCACGCAGAGTACACGCG	CAACCCCTGGATGCATCTGGA
**siRNA**	**Forward sequence (5′–3′)**	**Reverse sequence (5′–3′)**
hs-DYRK1A-si-1	GGGAGACGAUUCUAGUCAUdTdT	AUGACUAGAAUCGUCUCCCdTdT
hs-DYRK1A-si-2	GGUUUACAAUGAUGGUUAUdTdT	AUAACCAUCAUUGUAAACCdTdT
ms-DYRK1A-si	GAUUCAGCAACCUCUAACUAATT	UUAGUUAGAGGUUGCUGAAUCTT
Negative control	GGCUCUAGAAAAGCCUAUGCdTdT	dTdTCCGAGAUCUUUUCGGAUACG
**RNA oligo**	**Sense (5′–3′)**	**Antisense (5′–3′)**
Hs-miR-221-3p mimic	AGCUACAUUGUCUGCUGGGUUUC	AACCCAGCAGACAAUGUAGCUUU
Negative control	UUCUCCGAACGUGUCACGUTT	ACGUGACACGUUCGGAGAATT
