# Supplementary material for: MicroRNA-221-3p inhibits the inflammatory response of keratinocytes by regulating the DYRK1A/STAT3 signaling pathway to promote wound healing in diabetes
Source: Commun Biol. 2024 Mar 9;7:300. doi: 10.1038/s42003-024-05986-0 (PMC10924844; doi:10.1038/s42003-024-05986-0)
Supplement: Supplementary file 5 — Reporting Summary [file 42003_2024_5986_MOESM5_ESM.pdf]

nature portfolio

Corresponding author(s): COMMSBIO-23-0895B
Qiu Zhang

Last updated by author(s): Feb 25, 2024

Reporting Summary

Nature Portfolio wishes to improve the reproducibility of the work that we publish. This form provides structure for consistency and transparency in reporting. For further information on Nature Portfolio policies, see our Editorial Policies and the Editorial Policy Checklist.

Statistics

For all statistical analyses, confirm that the following items are present in the figure legend, table legend, main text, or Methods section.

n/a	Confirmed	
☐	☒	The exact sample size (*n*) for each experimental group/condition, given as a discrete number and unit of measurement
☐	☒	A statement on whether measurements were taken from distinct samples or whether the same sample was measured repeatedly
☐	☒	The statistical test(s) used AND whether they are one- or two-sided<br>*Only common tests should be described solely by name; describe more complex techniques in the Methods section.*
☒	☐	A description of all covariates tested
☒	☐	A description of any assumptions or corrections, such as tests of normality and adjustment for multiple comparisons
☐	☒	A full description of the statistical parameters including central tendency (e.g. means) or other basic estimates (e.g. regression coefficient) AND variation (e.g. standard deviation) or associated estimates of uncertainty (e.g. confidence intervals)
☐	☒	For null hypothesis testing, the test statistic (e.g. *F*, *t*, *r*) with confidence intervals, effect sizes, degrees of freedom and *P* value noted<br>*Give P values as exact values whenever suitable.*
☒	☐	For Bayesian analysis, information on the choice of priors and Markov chain Monte Carlo settings
☒	☐	For hierarchical and complex designs, identification of the appropriate level for tests and full reporting of outcomes
☒	☐	Estimates of effect sizes (e.g. Cohen's *d*, Pearson's *r*), indicating how they were calculated

Our web collection on statistics for biologists contains articles on many of the points above.

Software and code

Policy information about availability of computer code

Data collection	No software was used.
Data analysis	Data analysis conducted using GraphPad Prism software (GraphPad Software, Inc. La Jolla, California). Western blotting analysis was performed using ImageJ Software.

For manuscripts utilizing custom algorithms or software that are central to the research but not yet described in published literature, software must be made available to editors and reviewers. We strongly encourage code deposition in a community repository (e.g. GitHub). See the Nature Portfolio guidelines for submitting code & software for further information.

Data

Policy information about availability of data

All manuscripts must include a data availability statement. This statement should provide the following information, where applicable:

- Accession codes, unique identifiers, or web links for publicly available datasets
- A description of any restrictions on data availability
- For clinical datasets or third party data, please ensure that the statement adheres to our policy

The data underlying this article will be shared on reasonable request to the corresponding author. The original RNA-seq data of this study has been uploaded to public data (Sequence Read Archive: PRJNA956529). BioProject and associated SRA metadata are available at https://dataview.ncbi.nlm.nih.gov/objectPRJNA956529?reviewer=mujplpco4lit1939h02d8kltu5 in read-only format.

Human research participants

Policy information about studies involving human research participants and Sex and Gender in Research.

Reporting on sex and gender	All participants were males aged between 40 and 60 years
Population characteristics	All participants were males aged between 40 and 60 years
Recruitment	Human samples were collected at the Department of Endocrinology of The First Affiliated Hospital of Anhui Medical University.
Ethics oversight	All clinical experiments were approved by the ethics committee.

Note that full information on the approval of the study protocol must also be provided in the manuscript.

Field-specific reporting

Please select the one below that is the best fit for your research. If you are not sure, read the appropriate sections before making your selection.

☒ Life sciences ☐ Behavioural & social sciences ☐ Ecological, evolutionary & environmental sciences

For a reference copy of the document with all sections, see nature.com/documents/nr-reporting-summary-flat.pdf

Life sciences study design

All studies must disclose on these points even when the disclosure is negative.

Sample size	Sample size were provided in the manuscript. The sample sizes of clinical samples and animal experiment were based on experience and existing literature.
Data exclusions	No data were excluded from the analyses.
Replication	All experiments were run over triplicate to verify the reproducibility of experimental findings.
Randomization	All mice were randomly distributed into groups.
Blinding	All experiments in mice were done and analyzed blind,and clinical samples were collected in strict accordance with diagnostic criteria

Reporting for specific materials, systems and methods

We require information from authors about some types of materials, experimental systems and methods used in many studies. Here, indicate whether each material, system or method listed is relevant to your study. If you are not sure if a list item applies to your research, read the appropriate section before selecting a response.

Materials & experimental systems

n/a	Involved in the study	
☐	☒	Antibodies
☐	☒	Eukaryotic cell lines
☒	☐	Palaeontology and archaeology
☐	☒	Animals and other organisms
☒	☐	Clinical data
☒	☐	Dual use research of concern

Methods

n/a	Involved in the study	
☒	☐	ChIP-seq
☐	☒	Flow cytometry
☒	☐	MRI-based neuroimaging

Antibodies

Antibodies used	The antibodies used were anti-DYRK1A (DF3270, Affinity Biosciences, China), anti- phospho-STAT3 (Tyr705) (9145S, Cell Signaling Technology, USA), anti- phospho-STAT3 (Ser727) (9134S, Cell Signaling Technology, USA), anti-STAT3 (9139S, Cell Signaling Technology, USA), anti-β-actin/anti-β-tubulin (AF7018/AF7011, Affinity Biosciences, China), and anti-rabbit IgG (A7016, Beyotime Biotechnology, China).
Validation	The validation of commercial antibodies used in this study were posted in manufacturer's website.

Eukaryotic cell lines

Policy information about cell lines and Sex and Gender in Research

Cell line source(s)	Human immortal keratinocyte line (HaCaT)
Authentication	Cell line used in the study were authenticated by CLASTR (https;//web.wxpasy.org/cellosaurus-str-search/).
Mycoplasma contamination	Cell line testes negative for mycoplasma contamination.
Commonly misidentified lines (See ICLAC register)	N/A

Animals and other research organisms

Policy information about studies involving animals; ARRIVE guidelines recommended for reporting animal research, and Sex and Gender in Research

Laboratory animals	All C57BL/6 WT mice and Mir221 KO mice were male and 6-8 weeks of age.
Wild animals	No wild animals were involved in the study.
Reporting on sex	Only male mice were included in this study.
Field-collected samples	No samples were collected from the field in the study.
Ethics oversight	All animal experiments were approved by the local authorities and performed in accordance with the guidelines of the Animal Care and Use Committee of Anhui Medical University.

Note that full information on the approval of the study protocol must also be provided in the manuscript.

Flow Cytometry

Plots

Confirm that:

☒ The axis labels state the marker and fluorochrome used (e.g. CD4-FITC).

☒ The axis scales are clearly visible. Include numbers along axes only for bottom left plot of group (a 'group' is an analysis of identical markers).

☒ All plots are contour plots with outliers or pseudocolor plots.

☒ A numerical value for number of cells or percentage (with statistics) is provided.

Methodology

Sample preparation	Human primary neutrophils were isolated from 0.2% EDTA-anticoagulated whole blood collected by venipuncture from a healthy donor. Erythrocytes were removed using Red Blood Cell Lysis Buffer (Tiangen Biotech, China). Isolated cells were stained with anti-human CD66b (305104, Biolegend, China) and anti-human CD16 (302008, Biolegend) for 30 min at 4 °C.
Instrument	Flow cytometry was performed using a Moflo-XDP system (Beckman Coulter).
Software	FlowJo V10.
Cell population abundance	only double-stained white cells were isolated (anti-human CD66b (305104, Biolegend, China) and anti-human CD16 (302008, Biolegend).
Gating strategy	Gating strategy that inviolved in this study are openly available from the corresponding author upon any request. If necessary, we will add the gating strategy figures in supplementary materials.

☒ Tick this box to confirm that a figure exemplifying the gating strategy is provided in the Supplementary Information.
